# Compound genetically engineered mouse models of cancer reveal dual targeting of ALK1 and endoglin as a synergistic opportunity to impinge on angiogenic TGF-β signaling

**DOI:** 10.18632/oncotarget.12604

**Published:** 2016-10-12

**Authors:** Nikolas M. Eleftheriou, Jonas Sjölund, Matteo Bocci, Eliane Cortez, Se-Jin Lee, Sara I. Cunha, Kristian Pietras

**Affiliations:** ^1^ Division of Translational Cancer Research, Department of Laboratory Medicine, Lund University, Medicon Village, Lund, Sweden; ^2^ Department of Molecular Biology and Genetics, Johns Hopkins University School of Medicine, Baltimore, MD, USA; ^3^ Department of Immunology, Genetics and Pathology, Uppsala University, Uppsala, Sweden

**Keywords:** angiogenesis, targeted therapy, BMP9, ALK1, endoglin

## Abstract

Angiogenesis occurs early in tumor development, sustains primary tumor growth and provides a route for metastatic escape. The TGF-β family receptors modulate angiogenesis via endothelial-cell specific pathways. Here we investigate the interaction of two such receptors, ALK1 and endoglin, in pancreatic neuroendocrine tumors (PanNET). Independently, ALK1 and endoglin deficiencies exhibited genetically divergent phenotypes, while both highly correlate to an endothelial metagene in human and mouse PanNETs. A concurrent deficiency of both receptors synergistically decreased tumor burden to a greater extent than either individual knockdown. Furthermore, the knockout of *Gdf2* (BMP9), the primary ligand for ALK1 and endoglin, exhibited a mixed phenotype from each of ALK1 and endoglin deficiencies; overall primary tumor burden decreased, but hepatic metastases increased. Tumors lacking BMP9 display a hyperbranching vasculature, and an increase in vascular mesenchymal-marker expression, which may be implicit in the increase in metastases. Taken together, our work cautions against singular blockade of BMP9 and instead demonstrates the utility of dual blockade of ALK1 and endoglin as a strategy for anti-angiogenic therapy in PanNET.

## INTRODUCTION

Induction of neo-angiogenesis is a compulsory hallmark of cancer and an early event during tumor progression [1]. Substantial efforts to develop angiogenesis inhibitors to treat cancer have resulted in a set of clinically approved drugs with blockade of vascular endothelial growth factor (VEGF) signaling as a common mechanism of action [2, 3]. Despite the fact that VEGF inhibitors are included in the first-line therapy against advanced and metastatic cancer of the colon, kidney, lung, liver and neuroendocrine pancreas, among others, the search for alternative and/or complementary targets for drug development is highly warranted due to a lack of persistent efficacy or substantial improvements of overall survival with currently used compounds.

The transforming growth factor (TGF)-β family consists of more than 30 ligands that bind and signal through serine/threonine kinase receptors (TGF-β type I and type II receptors) and accessory transmembrane proteins (TGF-β type III receptors) [4]. Evidence for a profound role for TGF-β signaling in angiogenesis comes from studies demonstrating that family ligands, such as TGF-β and bone morphogenetic protein (BMP) 9, activate receptor complexes on many cell types relevant to angiogenesis, including endothelial cells and perivascular cells. Moreover, genetic ablation of various receptors or ligands from the TGF-β family, most notably the TGF-β type I receptors activin receptor-like kinase (ALK)1 and ALK5, results in embryonic lethality due to vasculogenic or angiogenic defects [5]. In addition, gene knock-out for endoglin, an endothelial-cell selective TGF-β type III receptor, gives rise to a phenotype with close similarities to that of ALK1 ablation, in line with their analogous expression patterns, similar upregulation during active angiogenesis both in development and pathological conditions, and causal role in the genetic vascular deficiency syndrome hereditary hemorrhagic telangiectasia [6, 7]. However, the precise role for these signaling partners and their ligands during the complex process of angiogenesis has proven difficult to pinpoint, as their actions appear highly concentration and context dependent [8].

In the setting of tumor angiogenesis, genetic or pharmacologic targeting of ALK1 (gene name *Acvrl1*) results in a significant growth delay and angiogenic blockade [9–13]. In addition, ALK1 inhibition substantially impacts on metastatic dissemination by reducing the colonization of distant organs in a range of mouse models of cancer, including mammary carcinoma and pancreatic neuroendocrine tumor (PanNET)[12]. Conversely, in our comparative studies, genetic targeting of endoglin (gene name *Eng*), either globally or in an endothelial cell-restricted manner, only transiently impacts on tumor growth and angiogenesis [14]. Surprisingly, a reduced dosage of the endoglin gene gives rise to an increase in metastatic spread due to adoption of a mesenchymal phenotype by endothelial cells, which in turn leads to enhanced tumor cell intravasation [14, 15]. The divergent results on tumor parameters following targeting of ALK1 or endoglin, both thought to act in concert in the same endothelial cell regulatory pathway, begs the questions of which signaling event is dominant and whether combinatorial targeting would be beneficial. Also, it is still unclear what role BMP9, the most prominent ligand for both ALK1 and endoglin, plays in the regulation of tumor angiogenesis through its receptors.

Herein, we have aimed to further elucidate the functional dependence between ALK1 and endoglin in determining hallmark capabilities of tumorigenesis through targeting studies in compound genetically engineered mice. Furthermore, we have utilized mice deficient for BMP9 (gene name *Gdf2*) to investigate the impact of ligand binding to ALK1 and endoglin on tumor parameters. Our studies demonstrated a specific correlation of the expression of *ACVRL1* and *ENG* with an endothelial metagene in human PanNETs. In mechanistic studies, combined deficiency for one allele each of *Acvrl1* and *Eng* resulted in severe retardation of the development of experimental PanNETs in mice, in conjunction with suppression of angiogenesis and metastatic dissemination. In contrast, despite reducing tumor volume, deficiency for *Gdf2* gave rise to an enhanced incidence of micrometastatic lesions in the liver. Taken together, we have demonstrated the utility of combinatorial targeting of TGF-β family signaling to impair tumor growth and metastatic dissemination, although caution is warranted in the choice of target molecule.

## RESULTS

### Concomitant *Acvrl1* and *Eng* deficiency synergistically decreases pancreatic neuroendocrine tumor volume

**Figure 1:**
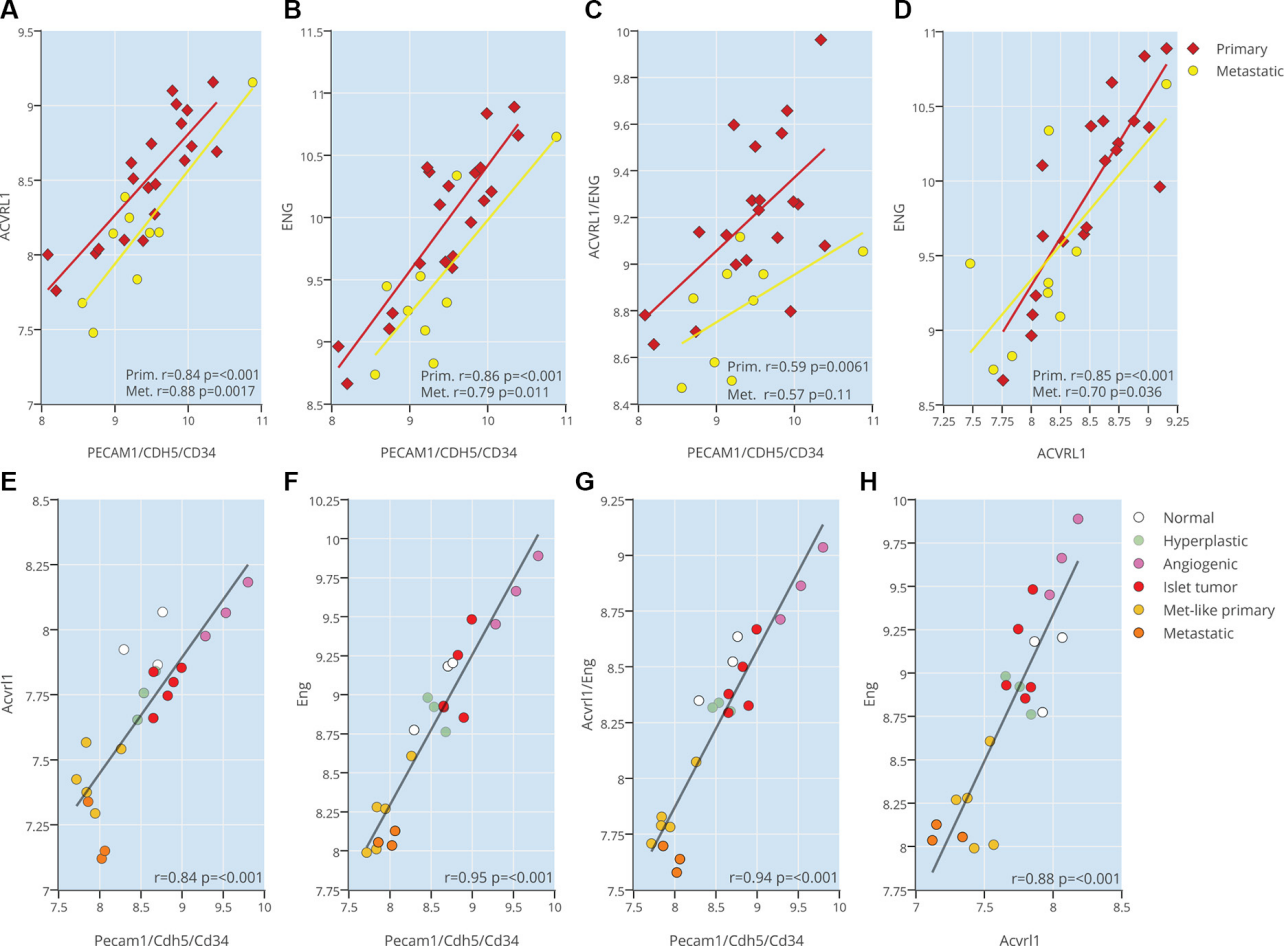
ACVRL1 and ENG expression are correlated to tumor neovasculature (**A**–**C**) Expression correlation of ACVRL1 and ENG against an endothelial metagene (*CD34*, *CDH5*, *PECAM1*) (A–C), and against each other (**D**) from a human dataset of pancreatic neuroendocrine tumors and metastases (**E**–**H**) Expression correlation of *Acvrl1* and *Eng* against an endothelial metagene (*Cd34*, *Cdh5*, *Pecam1*) (A-C), and against each other (D) from a mouse dataset of pancreatic neuroendocrine RIP1-TAg2 islets, tumors and metastases.

Our previous studies indicate that genetic ablation of *Acvrl1* or *Eng* in the context of pancreatic neuroendocrine tumorigenesis in RIP1-TAg2 mice [16] gives rise to divergent phenotypes, despite the fact that the two receptors both bind the predominant ligand BMP9, but also TGF-β [9, 12, 14]. The apparent contextual manner in which TGF-β family signaling functions depending on the activity of other receptors and/or ligands, led us to further investigate the extent of interaction within the endothelial TGF-β signaling pathways. Analysis of the expression of *ACVRL1* and *ENG*, either alone or combined, in 20 human PanNETs and 9 metastases (cohort previously reported in [17]) was demonstrated to be highly correlated to an endothelial cell metagene consisting of *CD34*, *CDH5* and *PECAM1* (Figure 1A–1C), indicating an exclusive endothelial cell expression within the tumor neovasculature. In addition, the expression of *ACVRL1* and *ENG* were significantly correlated to each other, suggesting that the two receptors act in concert (Figure 1D). The abundance of transcript for *ACVRL1* and *ENG* in primary tumors and metastatic lesions was similar (Figure 1A–1D). All correlations were confirmed in mouse PanNETs from RIP1-TAg2 mice, where expression of *Acvrl1* and *Eng* was found to be highest during the angiogenic phase of tumor development, compared to pre-malignant lesions (normal or hyperplastic islets), primary tumors (islet tumors or metastasis-like primary tumors), or hepatic metastases (Figure 1E–1H).

**Figure 2:**
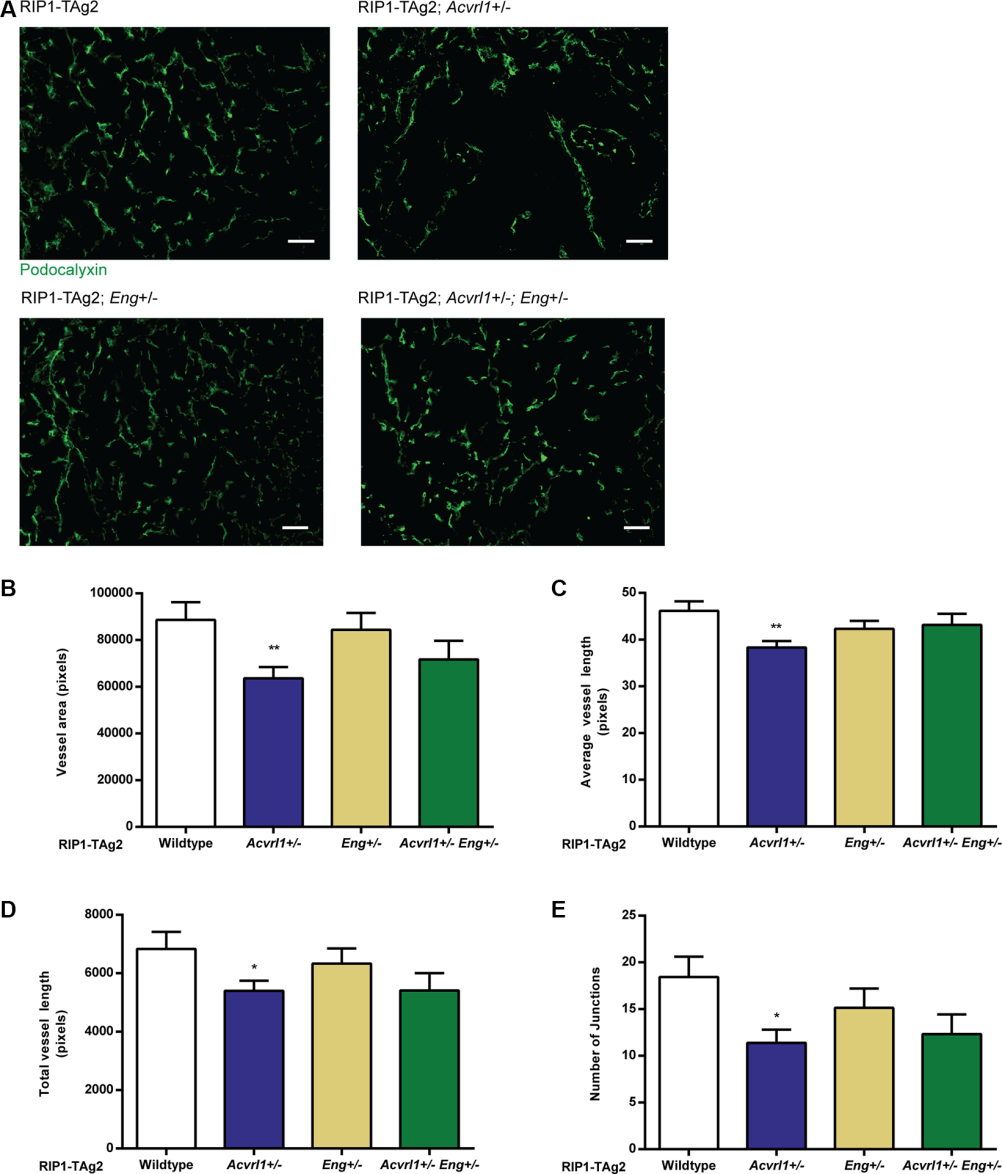
Deficiency of Acvrl1 alone reduces tumor angiogenesis (**A**) Representative images of Podocalyxin-stained vessels in PanNETs of 12-week old RIP-TAg2 mice. Scale bar 50 μm. B-E Vessel analysis of Podocalyxin-stained vessels in PanNETs of 12-week old RIP-TAg2 mice; total vessel area (**B**), average vessel length (**C**), total vessel length (**D**), and total number of junctions (**E**). *n* = 2–3 mice Data are mean ± SEM. *P < 0.05; **P < 0.01 vs. Wildtype with Student's *t-test*

We next sought to explore the utility of a dual targeting strategy for *Acvrl1* and *Eng* by generating RIP1-TAg2 mice with loss of one copy of each gene. While the global homozygous knockout of *Acvrl1* or *Eng* is each embryonic lethal due to vascular malformations [6, 18], the double heterozygous deficient mice (*Acvrl1*^+/−^*Eng*^+/−^) were viable, fertile, generated offspring in Mendelian ratios and did not display any obvious impairment in growth. Our previous studies have explored the individual heterozygous deficiencies in RIP-TAg2 mice, showing that receptor expression decreases proportionately, and further describes consequential phenotypes in vascularity, dissemination and changes in downstream target gene induction [9, 14]. Vessels of PanNET in compound RIP1-TAg2 mice were visualized by immunostaining for the luminal endothelial cell marker podocalyxin. In line with our previous report [9], single deficiency for *Acvrl1* gave rise to a significantly reduced total vessel area, total vessel length and total number of junctions compared to PanNET from wildtype RIP1-TAg2 mice, characteristic of an overall impairment in angiogenesis (Figure 2A–2E). In addition, and as demonstrated by our previous study [14], deficiency for *Eng* did not impact on vessel parameters in PanNET from RIP1-TAg2 mice at 12 weeks of age (Figure 2A–2E). Double deficiency for *Acvrl1* and *Eng* reduced vessel area and length, but did not further exacerbate the phenotype of single *Acvrl1* deficiency (Figure 2A–2E).

**Figure 3:**
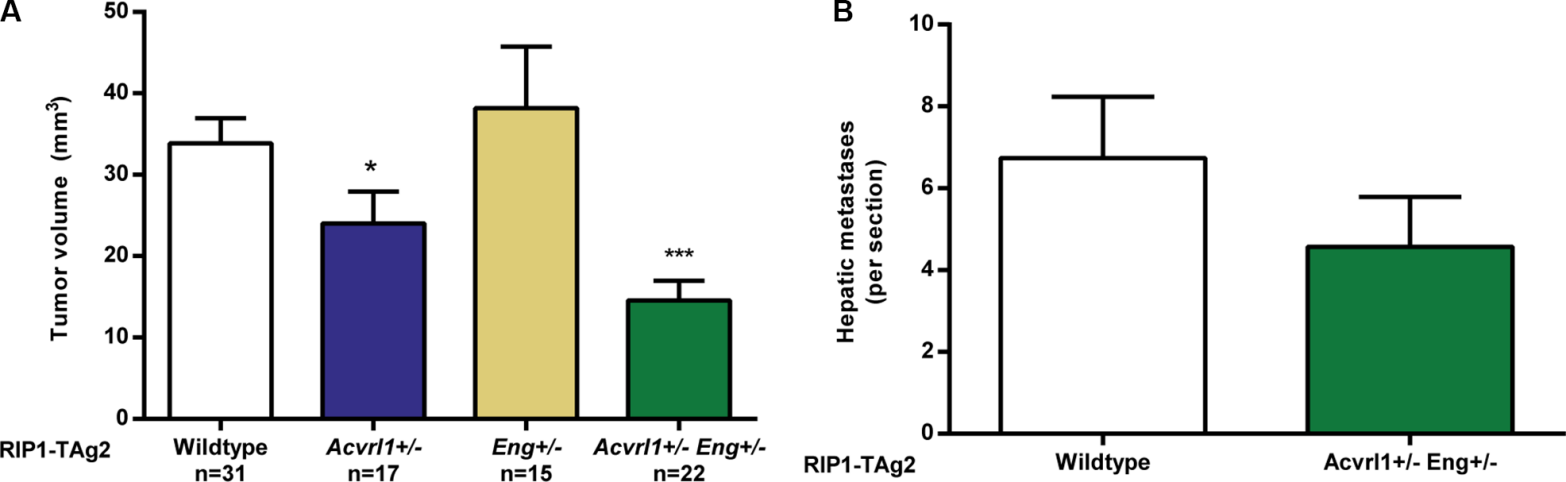
Acvrl1 and Eng deficiency synergistically decrease pancreatic neuroendocrine tumor volume (**A**) Total PanNET tumor volumes from RIP1-TAg2 wildtype, *Acvrl1*- and *Eng*-deficient mice at 12 weeks of age. (**B)** Number of individual liver micrometastases from RIP1-TAg2 mice, *n* = 5 mice per group Data are mean ± SEM. ***P < 0.001 vs. Wildtype with Student's *t-test*

In agreement with our previous reports, ablation of one copy of *Acvrl1* delayed tumor growth, while reducing the *Eng* gene dosage by half did not affect the growth rate of PanNET in RIP1-TAg2 mice (Figure 3A). Interestingly, 12-week old compound RIP1-TAg2; *Acvrl1*^+/−^*Eng*^+/−^ mice presented with a significantly reduced overall tumor burden by 57% and 39%, compared to RIP1-TAg2; *Acvrl1*^+/+^*Eng*^+/+^ mice or RIP1-TAg2; *Acvrl1*^+/−^*Eng*^+/+^ mice, respectively (Figure 3A). In addition, a trend towards fewer hepatic micrometastatic lesions was observed in RIP1-TAg2; *Acvrl1*^+/−^*Eng*^+/−^ mice compared to RIP1-TAg2; *Acvrl1*^+/+^*Eng*^+/+^ mice at 12 weeks of age (Figure 3B).

Taken together, our analyses of compound RIP1-TAg2; *Acvrl1*^+/−^*Eng*^+/−^ mice illustrates the utility of combinatorial targeting of ALK1 and endoglin in reducing hallmark parameters of tumor growth and progression.

### Ablation of BMP9 reduces the growth of primary PanNETs, while increasing the rate of metastasis

To understand the contribution of BMP9 ligand binding to the outcome of targeting each of its receptors ALK1 and endoglin, we made use of *Gdf2*-deficient mice. Knock-out mice for *Gdf2* are viable and fertile with no overt defect in blood vessel development, *e.g.* in neonatal vascularization of the retina, due to redundant signaling by the homologous ligand BMP10 [19]. However, *Gdf2*-deficient mice present with abnormal lymphatic vessel maturation and deficiency in lymphatic valve formation and lymph drainage, consistent with ALK1 expression in lymphatic endothelial cells [20, 21].

**Figure 4:**
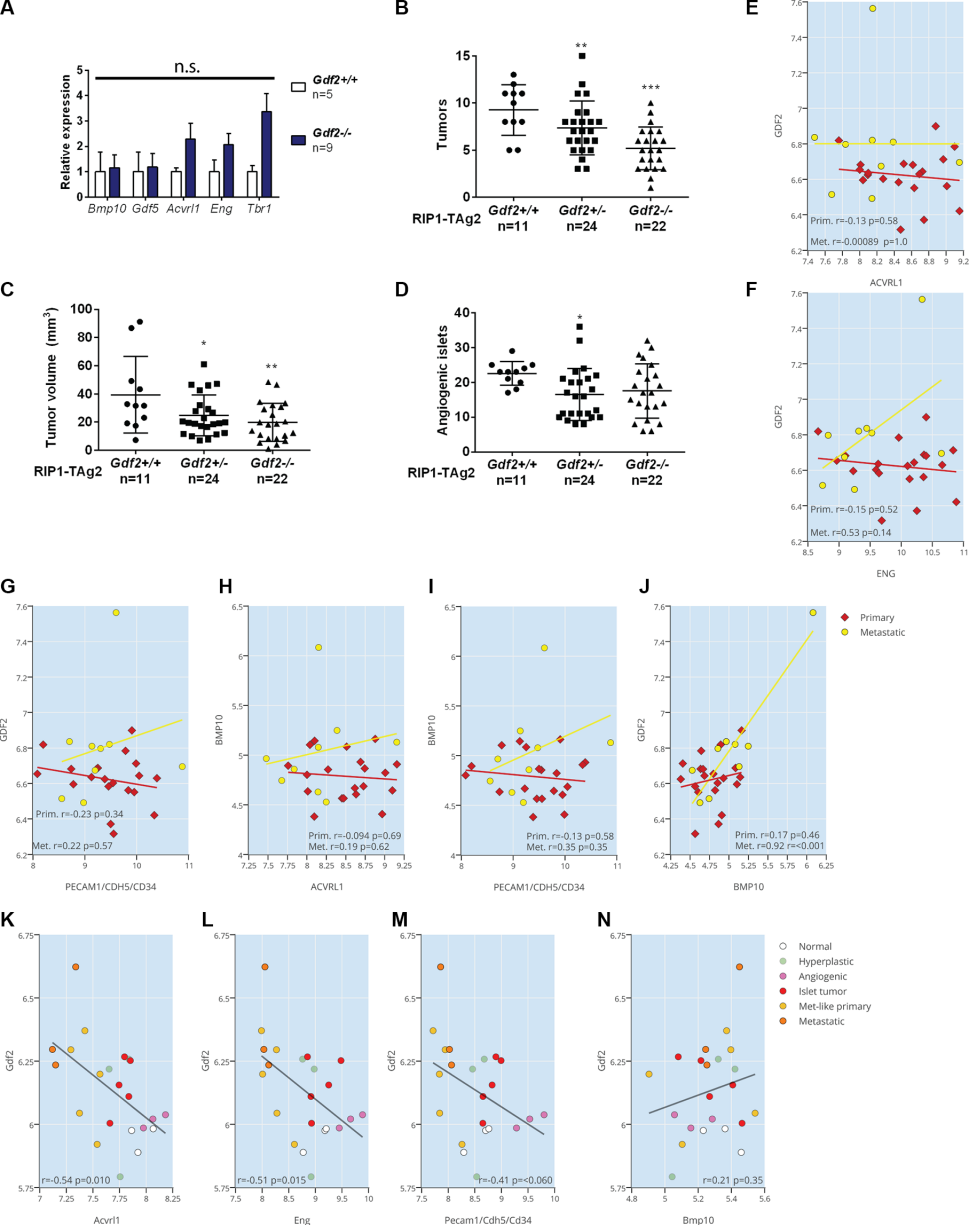
Ablation of BMP9 reduces the growth of primary PanNETs (**A**) qRT-PCR expression levels of ligands BMP10 and GDF5, and receptors ALK1, Endoglin and ALK5 in whole PanNET tumors from RIP1-TAg2 wildtype, and *Gdf2* knockout mice at 12 weeks. (**B**–**D**) Total PanNET tumor volume (B), number of tumors (C) and number of angiogenic islets (D) from RIP1-TAg2 mice at 12 weeks. (**E**–**J**) Expression correlation of *GDF2* and *BMP10* against ACVRL1, ENG and an endothelial metagene (CD34, CDH5, PECAM1), (E–I), and of *GDF2* against *BMP10* (J) from a human dataset of pancreatic neuroendocrine tumors and metastases (**K**–**N**) Expression correlation of *Gdf2* against *Acvrl1*, *Eng*, an endothelial metagene (*CD34*, *CDH5*, *PECAM1*), and Bmp10 (K-N) from a mouse dataset of pancreatic neuroendocrine RIP1-TAg2 islets, tumors and metastases Data are mean ± SEM. *P < 0.05; **P < 0.01; ***P < 0.001 vs. Wildtype with Student's *t-test*.

Here, we investigated whether the expression of the closely related family members BMP10 or GDF5 was altered in PanNETs from RIP1-TAg2; *Gdf2*^−/−^ mice, but no compensatory upregulation was found in the tumors in the absence of BMP9 (Figure 4A). Similarly, there were no statistically significant changes in the expression of *Acvrl1, Eng* or *Tgfbr1*, although a trend towards elevated levels of all genes was discerned (Figure 4A). Charting of the tumorigenic progression demonstrated that RIP1-TAg2; *Gdf2*^−/−^ mice presented with a 44% reduction in the number of tumors (Figure 4B, 5.2 ± 0.5 tumors /mouse in *Gdf2*-deficient mice, compared to 9.3 ± 0.8 for wildtype RIP1-TAg2 mice), and a 49% reduction in total tumor volume (Figure 4C, 19.9 ± 2.9 mm^3^/mouse in *Gdf2*-deficient mice, compared to 39.4 ± 8.2 for wildtype RIP1-TAg2 mice). The tumor volume and number for RIP1-TAg2; *Gdf2*^+\−^ mice were intermediate between the wildtype and knockout phenotypes, demonstrating a gene dosage effect (Figure 4B–4C). In contrast, the number of angiogenic islets was not dependent on *Gdf2* deficiency, indicating little influence of BMP9 signaling in activating the angiogenic switch (Figure 4D). In support of a lack of direct effect of BMP9, or the related ligand BMP10, in the regulation of angiogenic vessel growth within human primary or metastatic PanNETs, the expression of *GDF2*, *BMP10* (Figure 4E–4J) or *GDF5* (data not shown) was neither correlated to an endothelial cell metagene, nor to the expression of their receptors *ACVRL1* or *ENG*. Notably, *GDF2* and *BMP10* expression was significantly correlated to each other (Figure 4J). Finally, in mouse PanNETs from RIP1-Tag2 mice, the expression of *Gdf2* was even found to be inversely correlated to its receptors *Acvrl1* and *Eng*, as well as to the vascular metagene (Figure 4K–4N).

**Figure 5:**
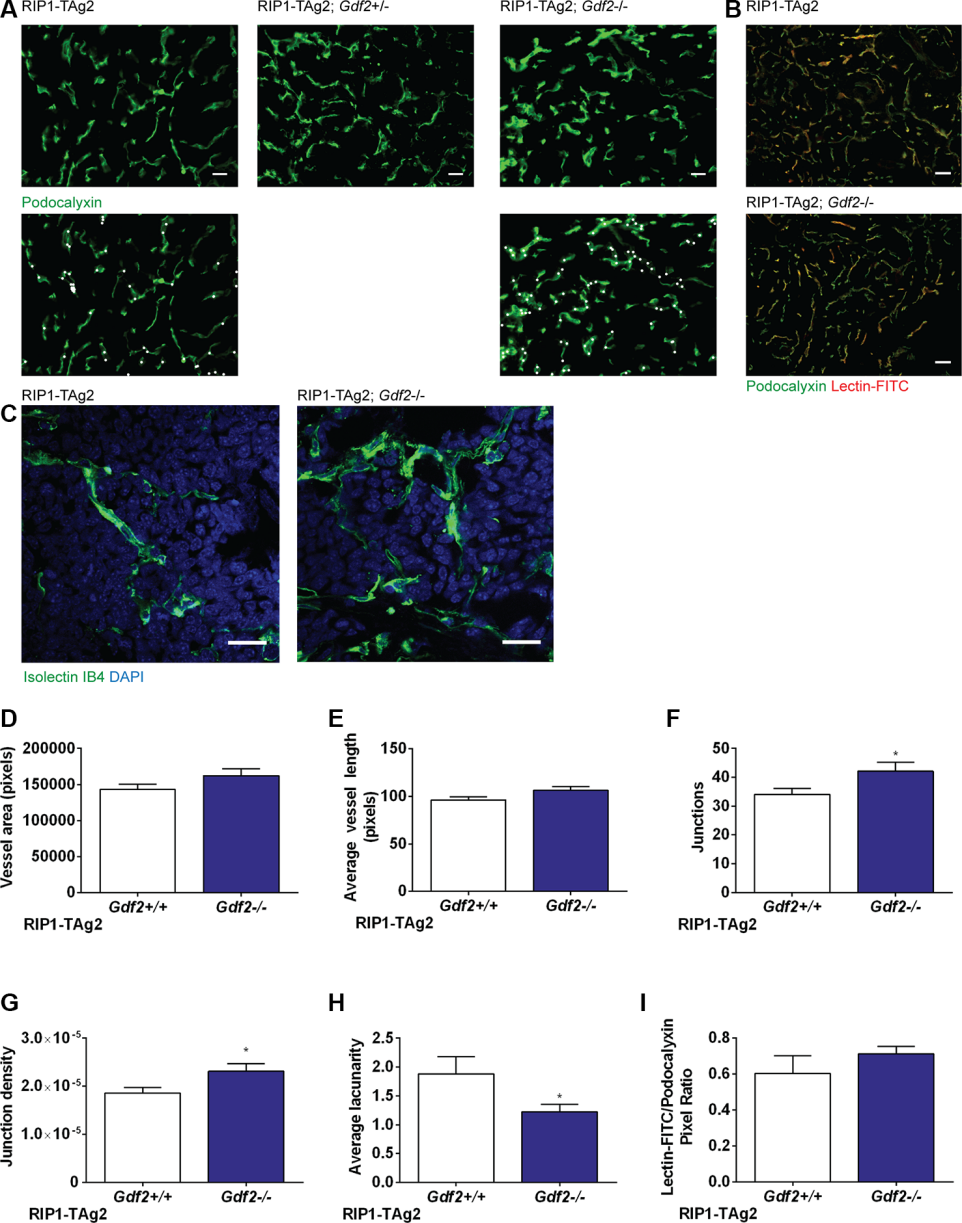
Ablation of BMP9 increases the number of vessel junctions and reduces lacunarity in primary tumor vasculature (**A**) Representative images of Podocalyxin-stained vessels in PanNETs of 12-week old RIP-TAg2 mice. Vessel junctions are highlighted in second row with white dots. Scale bar 20 μm. (**B**) Representative images of Lectin-FITC perfused vessels in PanNETs of 12-week old RIP-TAg2 mice. Scale bar 50 μm. (**C**) High-magnification vessel details in PanNETs stained with Isolectin GS-IB4. Scale bar 20 μm. (**D**–**H**) Analysis of vessel features in Podocalyxin-stained PanNETs from RIP1-TAg2 mice; total vessel area (C), average vessel length (D), total number of junctions (E), junction density (F), and lacunarity (G). (**I**) Quantification of immunofluorescence of FITC-conjugated Lectin perfusion relative to Podocalyxin expression. Data are mean ± SEM. *P < 0.05 vs. Wildtype with Student's *t-test*.

Next, to assess quantitative and qualitative differences in the angiogenic response due to the lack of BMP9, we performed a careful automated image analysis of the vessel tree, as detected by immunostaining for the endothelial cell marker podocalyxin and for tomato lectin used to visualize functional vessels (Figure 5A–5C). The total vessel area and the average vessel length were not affected by the absence of BMP9 (Figure 5D–5E). Notably, however, the number of vessel junctions and the branching density was significantly higher in tumors from *Gdf2*-deficient mice (Figure 5A–5G). In addition, the lacunarity of the vasculature, *i.e.* the irregularity and size of the gaps between blood vessels, was significantly lower in PanNET from RIP1-TAg2; *Gdf2*^−/−^ mice (Figure 5H). Finally, analysis of the proportion of functional vessels as visualized by injection with FITC-conjugated tomato lectin demonstrated no difference between groups (Figure 5I).

**Figure 6:**
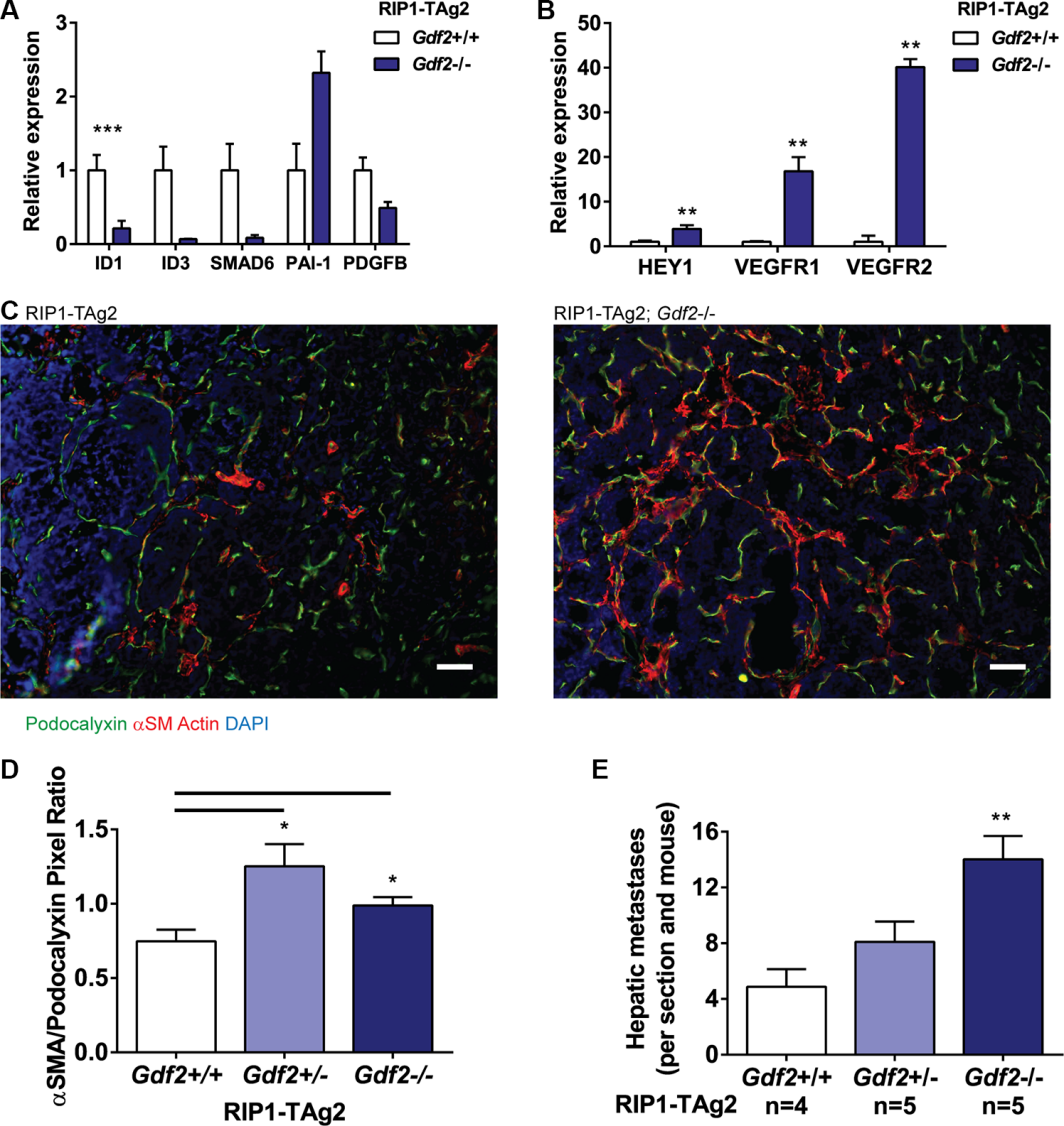
Ablation of BMP9 increases hepatic metastases and affects downstream ALK1 and Notch signaling (**A**–**B**) qRT-PCR expression levels of downstream products of ALK1 and ALK5 signaling (A), and Notch signaling (B) from LYVE1-/CD31+ endothelial cells isolated from PanNET tumors. Expression levels are relative to L19 housekeeping gene. (**C**) Representative images of labelled PanNET tumors from RIP1-TAg2 mice, showing Podocalyxin (green), α-Smooth Muscle Actin (red) and nuclei (DAPI, blue). Scale bar 50 μm. (**D**) Quantification of positively-stained pixels of α-Smooth Muscle Actin relative to Podocalyxin in PanNET tumors. *n* = 4 mice per group. (**E**) Number of individual hepatic micrometastases in RIP1-TAg2 mice at 12 weeks. SEM. *P < 0.05; **P < 0.01; ***P < 0.001 vs. Wildtype with Student's *t-test*.

Exploration of specific changes in gene expression in endothelial cells associated with deficiency for BMP9 demonstrated a dramatically impaired activity of the ALK1 signaling pathway, as assessed by the expression of the prototypical target genes *Id1*, *Id3* and *Smad6* in isolated PanNET endothelial cells, indicative of a non-redundant function for BMP9 in activating signaling in endothelial cells downstream of BMP receptors (Figure 6A). In contrast, the expression of ALK5 target genes in endothelial cells was inconsistently and relatively less regulated, effectively resulting in an increased ratio of ALK5/ALK1 activity (Figure 6A). ALK1 signaling has previously been implicated in the regulation of endothelial stalk cell/tip cell identity [19]. Intriguingly, the transcripts for both endothelial stalk cell-related genes, such as *Hey1* and *Flt1* (VEGFR1), and for the tip cell-related gene *Kdr* (VEGFR2), were increased in abundance in an isolated pool of endothelial cells from PanNET lesions in RIP1-TAg2; *Gdf2*^−/−^ mice, suggesting that both stalk cell and tip cell identity were reinforced in the absence of BMP9 (Figure 6B).

Finally, we characterized the support from vessel-associated mesenchymal cells by immunostaining for α-smooth muscle actin (α-SMA) in tumors from RIP1-TAg2 mice. Similar to *Eng*-deficient mice, RIP1-TAg2; *Gdf2*^−/−^ mice presented with PanNET harboring a greater investment of mesenchymal cells in the peri-vascular niche compared to their wildtype counterpart, with evidence for co-localization of podocalyxin and α-SMA, suggestive of ongoing EndMT (Figure 6C–6D). In our previous studies, loss of endoglin in the tumor endothelium was found to be functionally linked to a facilitated transmigration of malignant cells across the vessel wall, and thereby an increased metastatic seeding, through the process of EndMT [14]. Similarly, loss of BMP9, and the concomitantly intensified EndMT, was associated with a 66% rise in metastatic colonization of the liver in mice with one deficient copy of the *Gdf2* gene, and a 188% rise in the incidence of hepatic metastases in mice with ablation of both alleles of *Gdf2* (Figure 6E).

## DISCUSSION

**Figure 7:**
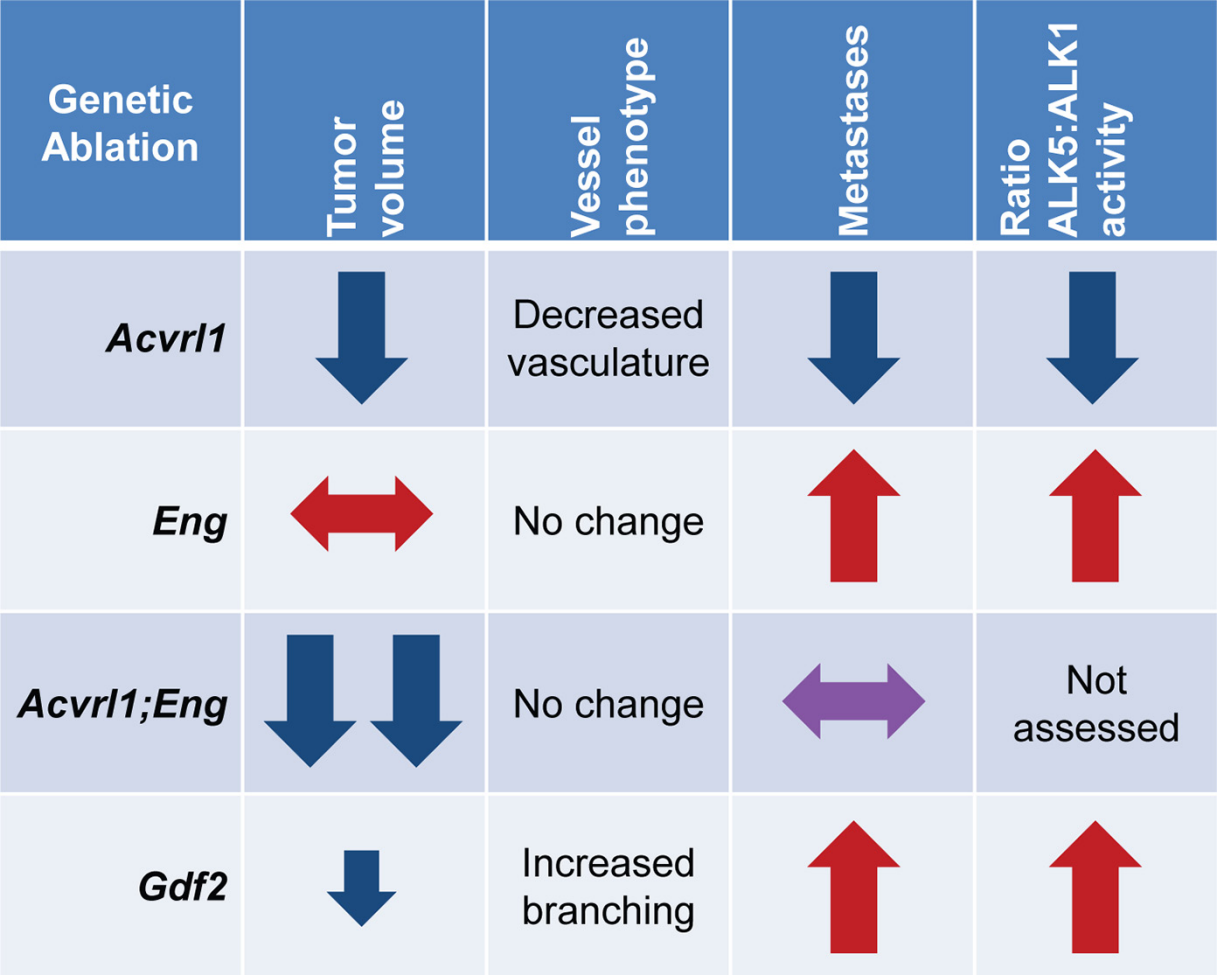
Representative RIP-TAg2 phenotypes for genetic ablation of *Acvrl1*, *Eng*, and *Gdf2* Red and blue arrows are representative of the *Acvrl1* and *Eng* deficient phenotypes, respectively.

A schematic representation of the contrasting phenotypes resulting from genetic perturbation of endothelial TGF-β family members in the context of the RIP1-TAg2 mouse model of PanNETs is shown in Figure 7. While targeting of ALK1 gives rise to suppression of key parameters, including tumor volume, vessel density and metastatic dissemination, suppression of endoglin enhances seeding of metastases [9, 12, 14]. Dual targeting of ALK1 and endoglin resulted in further inhibition of tumor growth, indicating that the most effective means to achieve repression of TGF-β signaling in endothelial cells is by blocking both receptors. Deficiency for BMP9 was accompanied by reduced growth of the primary tumor, in agreement with blockade of ALK1 signaling, combined with enhanced metastatic colonization, suggesting impairment of signaling via endoglin. The composite phenotype in RIP1-TAg2; *Gdf2*^-/-^ mice also implies that the inhibitory effect of ALK1 blockade cannot simply be explained by neutralization of BMP9. Thus, despite the fact that the activity of the canonical Smad1/5/8-mediated ALK1 pathway is mitigated in mice lacking BMP9, further studies to identify additional ligands that bind and activate atypical signaling through ALK1 may be warranted.

Our current and previous work shows that signaling by TGF-β family members in endothelial cells governs the rate of metastatic escape from the primary tumor. Deficiency for endoglin or BMP9 results in a shift towards a mesenchymal phenotype of endothelial cells, thus endorsing malignant cell transmigration across the vessel wall. In contrast, inhibition of ALK1 reduces EndMT in mouse models of breast cancer (unpublished observation). Endothelial-to-mesenchymal transition in endothelial cells is correlated to the activity of the ALK5 type I receptor pathway, such that during conditions of increased EndMT and metastatic spread (targeting of endoglin or BMP9), ALK5 target genes are increasingly transcribed compared to ALK1 target genes. Conversely, during conditions of reduced EndMT and impaired systemic dissemination (targeting of ALK1), ALK5 target genes exhibit a reduced expression. A recent study demonstrated stable complex formation between ALK1, endoglin and the TGF-β type II receptor, even in the absence of any ligand [22]. Interestingly, endoglin was shown to promote ALK1-dependent Smad1/5 signaling, consistent with our finding that reduced levels of endoglin shifts the balance towards the ALK5-induced Smad2/3 pathway and with previous studies demonstrating a requirement of endoglin for BMP9-induced ALK1 signaling [23]. Presumably, in the absence of ALK1 activity, endoglin may still sequester BMP9 and/or TGF-β from binding to ALK5, thereby explaining why ALK1 targeting does not promote mesenchymal transformation of the endothelium. The relative activation status of the ALK1 and ALK5 pathways appear to be an important determinant of the endothelial cell phenotype in relation to EndMT and endorsement of tumor cell transmigration; future studies should therefore explore this parameter as a potential biomarker of prognostic significance. Also, high resolution exploration of the complex formation between the different type I, type II and type III receptors of the TGF-β family expressed by endothelial cells should be pursued to determine the mechanism for their interdependence of activity.

Canonical ALK1 signaling cooperates with Notch to suppress VEGF-driven sprouting and tip-cell formation during physiological angiogenesis [24]. Moreover, reduced ALK1 signaling by targeting of BMP9 and BMP10 induces a hypersprouting phenotype both *in vitro* and *in vivo*, resembling Notch inhibition [19, 24, 25]. Mechanistically, ALK1- and ALK5-mediated activation of Smad 2/3 is triggered by Notch to drive the stalk cell phenotype required for resolution of the angiogenic cascade [26]. The lack of BMP9 in the context of physiological angiogenesis is moderated to some extent by upregulation of BMP10 [19, 27]. In contrast, in our studies of pathological tumor angiogenesis, we did not observe a compensatory increase in expression of BMP10 (or the close family member GDF5) within tumor lysates. However, while the absence of BMP9 did cause significant hyperbranching, it did not reduce vessel density to the same extent as targeting of ALK1, again suggesting activation of ALK1 by alternative ligand(s) or by compensatory production of ligands at distant sites. Whether the increased density of vessel branches in tumors from RIP1-TAg2; *Gdf2*^−/−^ mice is consequential to enhanced sprouting, similar to the situation during developmental angiogenesis, remains to be determined, but appears plausible. Our findings illustrate the added complexity of tumor angiogenesis compared to physiological angiogenesis, and the need for more refined studies of the intricate mechanisms of blood vessel growth within the malignant tissue.

There are currently several anti-cancer drugs targeting TGF-β family signaling, including dalantercept and PF-03446962 blocking ALK1, and TRC-105 neutralizing endoglin, in Phase II/III of clinical development for a range of indications [28–31]. Our comprehensive charting of the outcome of targeting ALK1, endoglin or BMP9 to achieve anti-angiogenic and therapeutic efficacy highlights the importance of performing detailed mechanistic studies prior to commencing clinical testing of drugs presumably impinging on the same signaling pathway, since the outcome may be diverse even when targeting closely related proteins. Specifically, our studies suggest that ALK1 would be the target of choice for the TGF-β signaling system in endothelial cells to best reduce angiogenesis [9, 12, 14]. Nevertheless, drugs for cancer indications are rarely used as monotherapy, therefore targeting of endoglin and/or BMP9 may still be pursued as partners in combinatorial treatments. Indeed, several phase II clinical trials are already in motion for combinations of inhibitors of TGF-β family signaling and well-established VEGF-targeting drugs, some of which have presented promising interim analyses [32]. The added value of combining anti-angiogenic therapies with different mechanisms of action is consistent with previous work, by us and others, identifying ALK1 or endoglin blockade as synergistic partners to VEGF inhibition in pre-clinical studies [11, 14]. Furthermore, the current study indicates dual targeting of ALK1 and endoglin as a promising additional possibility for combinatorial targeting of neoangiogenesis and tumor growth.

## MATERIALS AND METHODS

### Animal work and primary tumors

All animal experiments were approved by the ethical committees for animal care in Stockholm and Lund (permits N96/11 and M142/13, respectively). C57BL/6 RIP-TAg2 mice receive water supplemented with 5% sucrose at 10 weeks of age to counteract hypoglycemia. Mice were sacrificed at 12 weeks of age, and pancreatic neoplasms with diameter ≥ 1 mm were measured as tumors, and smaller angiogenic islets were quantified using a stereological microscope. The tumor burden was calculated as the total volumes of all tumors from the pancreas, where individual volumes were calculated as . length × 2width × π/6.

### Analysis of gene expression data

Human patient data (GSE73339) and RIP1-TAg2 data (GSE73514) were downloaded from the Gene Expression Omnibus (GEO) [17]. The human data set contained 20 primary and 9 metastatic PanNETs and the mouse PanNET data set consisted of a total of 22 samples (normal (*n* = 3), hyperplastic (*n* = 3), angiogenic (*n* = 3), islet tumor (*n* = 5), met-like primary (*n* = 5), and metastatic (*n* = 3) samples)). The data were accessed on January 8, 2016. Gene expression data generated by Sadanandam et al. [17] were obtained using the Affymetrix GeneChip human Gene 1.0 ST and Affymetrix GeneChip Mouse Gene 1.0 ST arrays and were normalized using RMA. Analysis was conducted with R Studio, and the Pearson method was used to analyze correlations. All *P-value*s reported are two-tailed.

### Immunofluorescence and histology

At time of sacrifice, mice were heart perfused with 10 mL each PBS and 4% paraformaldehyde or buffered zinc formalin. The left liver lobes were post-fixed in formaldehyde overnight at 4°C, paraffin embedded and serially sectioned. Each mouse contained 8 or 15 sections (Gdf2 and Acvril1/Eng experiments respectively) and 4–5 mice per group. Paraffin-embedded sections were de-paraffinized and stained with hemotoxylin and eosin, and metastatic foci were counted if they were at least > 6 cells in diameter. The pancreas and embedded tumors were cryopreserved; stored in 30% sucrose overnight at 4°C, then embedding in cryostat sectioning matrix. Cryosections were fixed in acetone, blocked with serum-free blocking solution (DAKO) for 90 min at ambient temperature and stained with primary antibody overnight at 4°C. Sections were then stained with Alexa fluorchrome 488 and 594 nm secondary antibodies (dilution 1:1000, Invitrogen) for 90 min at ambient temperature, and mounted with 4′,6-diamidino-2-phenylindole-containing media (Vector Laboratories). Primary antibodies used in this study were Podocalyxin (dilution 1:100; R&D Systems, AF1556), αSMA-Cy3 (Clone 1A4; dilution 1:100; Cy3 conjugated; Sigma, C6198), and Isolectin IB4 (dilution 1:50; AF488 conjugated; Thermo Fisher, I21411). Vessel perfusion was evaluated by retro-orbital injection of FITC-conjugated tomato lectin allowed to circulate for 4 min prior to sacrificed (50 μl at 0.5 mg/ml; Vector Laboratories), and sections subsequently stained with anti-podocalyxin and anti-FITC antibody (dilution 1:100, Thermo Fisher, 71–1900).

Immunofluorescence-stained sections were imaged and acquired using a Nikon Eclipse E800 microscope (Nikon Instruments), SPOT RTKE camera and SPOT advanced software (SPOT Imaging), or an Olympus BX63 microscope and DP80 camera and cellSens Dimension v 1.12 software (Olympus Corporation). Analysis of podocalyxin-positive vessel features in immunostained RIP-TAg2 tumors was performed using Angiotool semi-automated software [33], with 10 or more high-power images from each mouse. Quantification of αSMA and Lectin-FITC immunostaining was performed by quantifying positively-stained pixels relative to podocalyxin-stained pixels in Adobe Photoshop CS5.1. Confocal fluorescence imaging was performed with Zeiss LSM 710 system and ZEN Black software (Carl Zeiss AG).

### Endothelial cell isolation

RIP-TAg2 mice were perfused with PBS, and isolated PNET were finely cut and incubated with stirring in in 0.05 g collagenase type II (Worthington), 0.05 g collagenase IV (Invitrogen), 0.01 g DNase I (Sigma) for 15 min at 37°C with stirring. The mixture was passed through a 70-μm strainer, mixed with 20 mL ice-cold isolation buffer (DMEM supplemented with 0.2% BSA, 5% enzyme-free cell dissociation buffer [Gibco]), and centrifuged at 3400 rpm at 4°C for 5 min. The pellet was resuspended in 10 mL PharmLyse buffer and remained for 10 min at ambient temperature, and then added to 40 mL of DMEM-dissociation buffer. The pellet was resuspended in 1 ml isolation buffer and 50 μL Lyve-1 coated beads (eBiosciences 13-0443-82 ; CELLection biotin binder kit Invitrogen), and were incubated in an end-over-end shaker at 4°C for 45 mins. Magnetic separation was used, and the supernatant was then mixed and incubated with 50 μL CD31 coated beads (BD Pharmigen, 553371) Following magnetic separation and washing of the CD31+ fraction with isolation buffer, RNA isolation was completed using the RNeasy kit (Qiagen).

### Real-time qPCR

Preparation of cDNA was done using iScript cDNA Synthesis kit (Biorad). QPCR was done using a KAPA Sybr Fast qPCR kit (Kapa Biosystems) on an Agilent Technologies Stratagene Mx3005P, using the following primers: RPL19, ID1, ID3 PAI-1, PDGFB, CD31, VE-cadherin (Cunha et al., 2010), VEGFR2 (Hagberg et al., 2010), and SMAD6 (F: CCACCAACTCCCTCATCACT, R: CTGGTCGTACACCGCATAGA). The following pre-validated primers were obtained from Qiagen: RPL19 (Mm_Rpl19_2_SG), BMP10 (Mm_BMP10_1_SG), GDF5 (Mm_Gdf5_1_SG), HEY1 (Mm_Hey1_1_SG), VEGFR1 (Mm_Flt1_1_SG), and VEGFR2 (Mm_Kdr_1_SG).

### Statistical analysis

All measurements are depicted as mean ± SEM, and statistical analyses were performed using an unpaired two-tailed Student's *t* test. Statistical significance was considered using α = 0.05.
